# Reassessing Trabeculectomy: A Long-Term Study with Stringent Success Criteria

**DOI:** 10.3390/jcm13061629

**Published:** 2024-03-12

**Authors:** Philip Braun, Daniel Böhringer, Jens Jordan, Michael Reich, Philip Keye, Thomas Reinhard, Jan Lübke

**Affiliations:** 1 Eye Center, Medical Center, Faculty of Medicine, University of Freiburg, 79106 Freiburg, Germany; philip.braun@students.uni-freiburg.de (P.B.); daniel.boehringer@uniklinik-freiburg.de (D.B.); philip.keye@uniklinik-freiburg.de (P.K.); thomas.reinhard@uniklinik-freiburg.de (T.R.); 2 Augenärzte am Städel, 60596 Frankfurt, Germany; jens.jordan@uniklinik-freiburg.de (J.J.); michael.reich@uniklinik-freiburg.de (M.R.)

**Keywords:** glaucoma, trabeculectomy, mitomycin c, long-term efficacy

## Abstract

**Background:** The aim was to evaluate the long-term outcome and efficacy of primary trabeculectomy with adjunctive mitomycin c (MMC) for treating glaucoma. **Methods:** We examined the medical records of 286 eyes that underwent trabeculectomy between 2008 and 2009 at the University Eye Hospital in Freiburg, Germany. Preoperative and follow-up data were collected, including intraocular pressure (IOP) measurements, surgical glaucoma interventions, and prescribed glaucoma medication. The first success criterion was defined as IOP ≤ 15 mmHg with no use of pressure-lowering medication by the patient, the second criterion was defined as the absence of surgical revision, and the third criterion as no further IOP-lowering surgery excluding early revisions following trabeculectomy. Statistical analyses comprised Cox regression and Kaplan–Meier survival estimations. **Results:** The mean follow-up duration was 1841 days (5 years). The mean preoperative IOP was 26.1 mmHg. Evaluating the success criteria at the time of average follow-up yielded a success rate of only 25% for the first criterion but 80% for both the second and third success criteria. **Conclusions:** The findings suggest that trabeculectomy with adjunctive MMC can be an effective procedure for permanently lowering IOP. However, surgical revisions and/or further glaucoma surgeries might still be needed. The long-term success rate is lower in comparison to previous research, which may be explained by the stricter success criteria in our study.

## 1. Introduction

According to the World Health Organization (WHO) Vision report of 2019, 76 million people worldwide suffer from glaucoma. It is the second leading cause of blindness, with an increasing prevalence of 3.5% for people aged 40–80 years [1].

To the present day, intraocular pressure (IOP) remains the only modifiable risk factor for glaucoma [1,2]. Its treatment relies on three pillars: pharmacological therapy, laser trabeculoplasty, and surgery, with the latter acting as a last resort if the former two do not achieve satisfactory IOP reduction [1].

Since its inception by Cairns in 1968, trabeculectomy (TE) has been considered the gold standard for the surgical treatment of medically refractory glaucoma [3,4]. As with other filtration procedures, its challenge lies between the balance of episcleral tissue scarring and uncontrolled drainage causing hypotony [5]. The introduction of antimetabolites such as mitomycin c in the 1990s has helped to tackle the issue of scarring and improve pressure regulation [6].

Most studies have only assessed the mid-term efficacy of TE, combined with a limited number of patients enrolled and mild success criteria [5,7,8]. Furthermore, previous studies have shown that stricter IOP targets below 15 mmHg can be beneficial for managing glaucomatous damage in the long term [9,10]. The aim of this study is therefore to provide a longer follow-up period, larger patient cohort, and stricter success criteria to evaluate the long-term efficacy of the procedure.

## 2. Materials and Methods

We conducted a retrospective cohort study, analyzing patients who received TE with adjunctive MMC between January 2008 and December 2009 at the University Eye Hospital in Freiburg, Germany.

Only patients that received primary TE with MMC and suffered from primary open-angle glaucoma (POWG), pseudoexfoliative glaucoma (PEX), and normal-tension glaucoma (NTG) were included. Preoperative and follow-up data such as demographic data, measured IOP, surgical glaucoma interventions, prescribed glaucoma medication, the use of anticoagulation, and co-morbidities such as diabetes mellitus were extracted from the medical records and entered into a research database. All continuous variables were described by mean and standard deviation, whereas categorical variables were reported in absolute and relative frequencies.

All procedures were performed by the same surgeon, under a standard surgical protocol and under regional or general anesthesia. A fornix-based conjunctival flap was prepared, followed by the insertion of a 0.2 mg/mL MMC-soaked sponge into the conjunctival flap for 3 min. After intensive rinsing with balanced salt solution (BSS), a rectangular scleral flap of 3 × 3 mm with half scleral thickness was dissected. Next, the trabeculectomy and peripheral iridectomy were performed and the scleral flap closed with 10–0 nylon sutures. The conjunctival flap was closed with absorbable 7.0 vicryl sutures. Lastly, the functioning of the filtering bleb was assessed with the injection of BSS into the anterior chamber. Furthermore, the filtering bleb was postoperatively reviewed through slit lamp examinations and the measuring of intraocular pressure.

The primary endpoint of this study was surgical success, for which we defined three different success criteria:

Success criterion one was achieved when IOP was ≤15 mmHg and no pressure-lowering medication was used by the patient.

Success criterion two was defined as when no revision surgery had to be undertaken. Needling, resuturing of the conjunctiva and sclera, and the injection of viscoelastics were considered as revisions.

Success criterion three was achieved when no additional IOP-lowering surgery excluding revision surgery after trabeculectomy was undertaken during follow-up.

Lastly, in a fourth analysis, success rates after revisional surgery were examined. Failure criterion was the need for another surgical intervention during followup.

Failures occurred when the aforementioned success criteria were not met at the earliest mentioning in the medical records. An overview of the different success criteria can be found in Table 1. Secondary outcome measure was frequency of postoperative complications. These included bleb leakage, macular edema, development of aqueous misdirection, and hyphema.

Kaplan–Meier survival analysis was performed to estimate all the success criteria separately. This was completed to assess the long-term efficacy of the procedure. In addition, we fitted a Cox proportional hazards regression model to identify potential risk factors for failure. We included age, gender, glaucoma sub-type, diabetes mellitus (preoperatively diagnosed), preoperative IOP, and the number of preoperative pressure-lowering medications as co-variates. Statistical analyses were performed with R (R foundation for Statistical Computing). The study was approved by the local ethics committee of the University of Freiburg (vote no. 21-1634-retro).

## 3. Results

We included a total of 286 eyes that underwent TE with MMC. Of these, 164 were from females (57%) and 122 from male patients (43%), with a mean age of 69.8 years and a mean follow-up time of 1841 days (5 years). The study population comprised 217 eyes with primary open-angle glaucoma, 58 eyes with pseudoexfoliative glaucoma, and 11 eyes with normal-tension glaucoma. In addition, 54 eyes (19%) were subject to anticoagulation and 26 (9%) were preoperatively diagnosed with diabetes. The mean preoperative IOP was 26.1 mmHg (±7.6). Table 2 summarizes the descriptive statistics of our study sample.

Concerning postoperative complications, a total of thirty eyes suffered from bleb leakage, two eyes were diagnosed with macular edema, a further two with aqueous misdirection, and finally thirteen eyes with hyphema. A summary of these complications can be found in Table 3.

Kaplan–Meier survival analysis was used to evaluate the success criteria.

**Figure 1:**
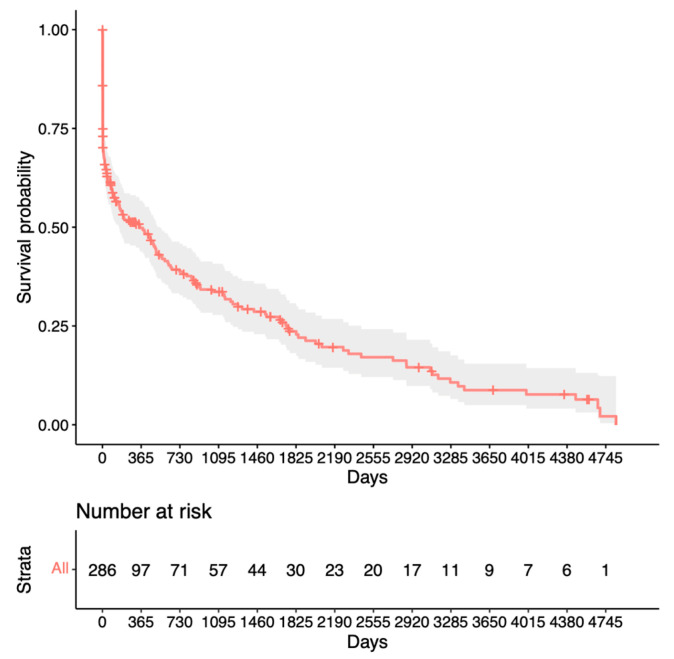
Kaplan–Meier survival analysis for the first success criterion.

The first success criterion group (IOP < 15 mmHg and no medication) is shown in Figure 1. The success rates were 55% after 1 year, 25% at the mean follow-up time, and 12.5% after 10 years (with only nine eyes at risk).

**Figure 2:**
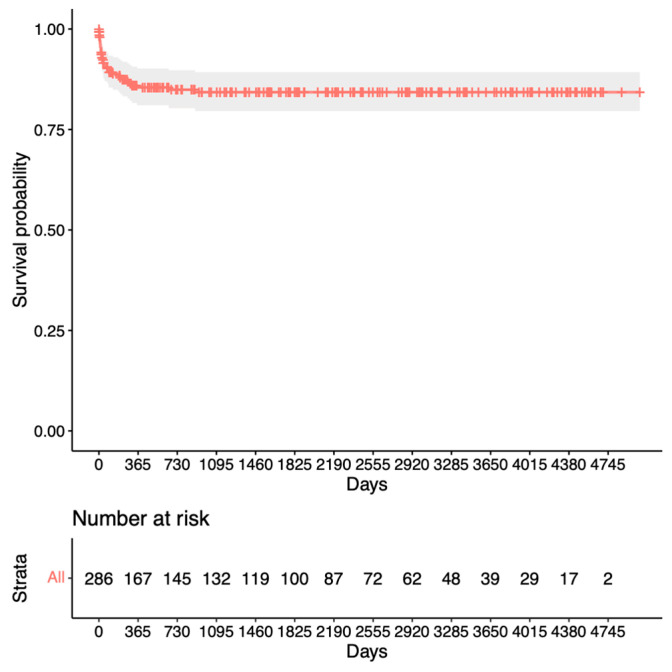
Kaplan–Meier survival analysis for the second success criterion.

Figure 2 presents the group for success criterion two (until revision surgery). The success rates were 85% at 1 year, 80% at the mean follow-up time, and unchanged after 10 years (with 39 eyes at risk).

**Figure 3:**
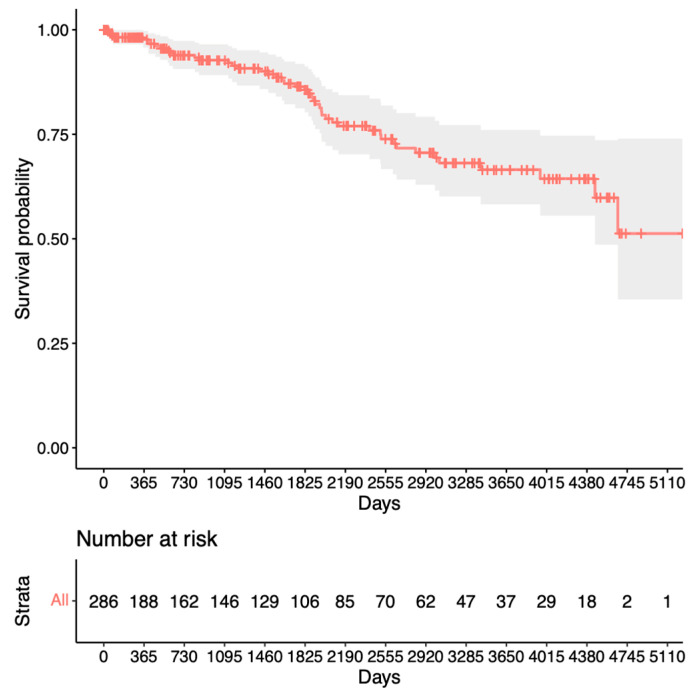
Kaplan–Meier survival analysis for the third success criterion.

The survival rates for the third success criterion group (until follow-up surgery) can be viewed in Figure 3. The survival rates after 1 year were 95%, after mean follow-up time 80%, and 70% after 10 years (with 37 eyes at risk).

**Figure 4:**
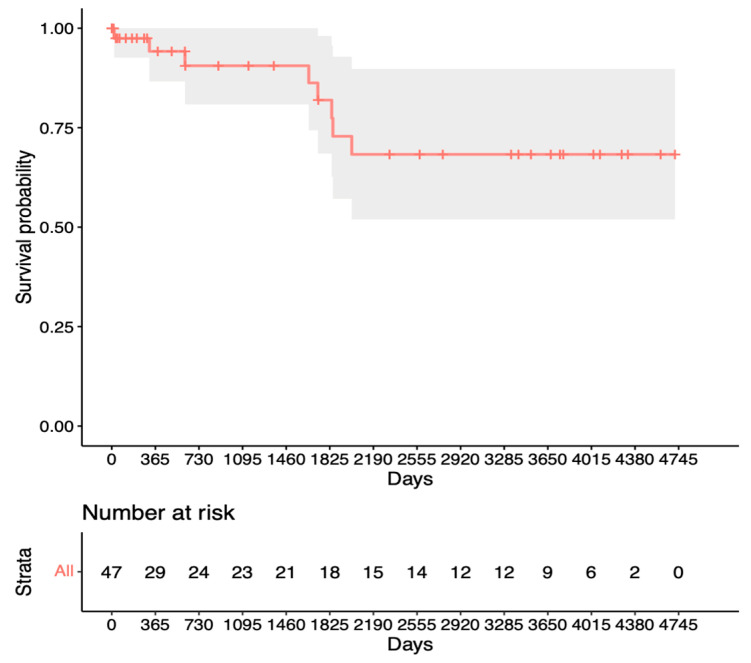
Kaplan–Meier survival analysis for the fourth success criterion.

Finally, Figure 4 shows the Kaplan–Meier analysis for success in terms of no other IOP-lowering surgery after revision surgery. It can be observed that the success rates were 90% at 1 year, 75% at the mean follow-up time, and 60% after 10 years (with only nine eyes at risk).

In addition, a Cox proportional hazards regression model was fitted in order to identify the risk factors for failure during the follow-up period. These factors included age, gender, glaucoma sub-type, preoperatively diagnosed diabetes, preoperative IOP, and the number of preoperative pressure-lowering medications. Of these, none turned out statistically significant at the 5% level.

## 4. Discussion

This retrospective cohort study sought to evaluate the long-term surgical outcomes of mitomycin C-augmented trabeculectomy using relatively stringent success criteria.

We chose these three relatively stringent criteria because trabeculectomy is generally considered to be a treatment option for patients who require intraocular pressure below 15 mmHg and/or wish to eliminate the need for IOP-lowering medication. Therefore, it appears to be a useful approach to assess the feasibility of meeting these criteria in a real-life dataset of patients. For the 286 eyes included in this study, the first success criterion was achieved for 55% after 1 year and 25% after the mean follow-up time of 5 years. These survival rates are lower compared to other studies conducted on this topic.

In a study by Beckers et al., the efficacy of trabeculectomy with mitomycin c was evaluated over a 5-year period, yielding a success rate of 83.4% after the first year and 60% after 5 years [7]. Opposed to our study, which defined success as an IOP of ≤15 mmHg without the use of glaucoma medication, Beckers et al. assessed the two criteria independently. In addition, the mean preoperative IOP was lower (22.3 mmHg ± 9.3 mmHg), and the postoperative complication rate of 51.7% was higher than in our study.

In 2010, Lusthaus et al. conducted a study examining 60 eyes with a follow-up of 3 years [5]. They observed an absolute surgical success rate of about 50% for the first and second year and 33% for the third year after trabeculectomy, defining absolute success as an IOP ≤ 18 mmHg without the use of pressure-lowering medication. The mean preoperative IOP was 25.3 mmHg (range 8–45 mmHg), with half of the eyes displaying an IOP of <6 mmHg within the first 6 weeks after surgery and only one eye experiencing bleb leakage.

The study by Ehrnrooth et al. retrospectively analyzed 138 eyes with a mean follow-up of 3.5 years, reporting success rates comparable to our study [8]. Absolute success was achieved at 63% after 1 year, 54% after 2 years, and 40% at 4 years, defining success as an IOP ≤ 21 mmHg without additional therapy. Notably, their criteria were milder than ours, and, concerning the number of complications, no information was provided.

Diestelhorst et al. reported success rates of 61%, 53.4%, and 37.8% at 1, 2, and 5 years, respectively [11]. Their success criteria included an IOP < 21 mmHg, no decreased visual acuity, no increased visual field loss, and no additional glaucoma surgery. The mean preoperative IOP was 28.5 mmHg (±9.8), and postoperative complications were not included in their study.

In a retrospective cohort study by Landers et al., the performance of trabeculectomy was assessed over a 20-year period and involved 330 procedures on 234 patients [4]. Complete surgical success was defined as an IOP < 21 mmHg without additional pressure-lowering medication and was achieved by 87% after one year, with a decline of 1.6% for every year thereafter, reaching 57% after 20-year follow-up. The mean IOP at diagnosis for primary open-angle glaucoma and normal-tension glaucoma was 30 mmHg.

Reibaldi et al. conducted a study on the long-term efficacy of trabeculectomy with a 9-year follow-up [12]. Surgical success was declared at an IOP < 18 mmHg without medication and <14 mmHg in two separate analyses, achieving 90% and 85%, respectively, after a follow-up of 5 years, making comparison with other studies difficult.

Most of the above-mentioned studies present higher success rates than those obtained in our study yet also show large heterogeneity in success criteria and mean preoperative IOP. Therefore, in order to investigate the effect of milder success criteria, we also conducted an analysis using an IOP < 21 mmHg without therapy as a success criterion. Notably, we achieved rates of 70% after 1 year and 43% at mean follow-up, suggesting better results than all the other studies under consideration except for the studies by Beckers et al., Landers et al., and Reibaldi et al. Moreover, our study reported a lower overall complication rate, with the exception of bleb leakages in Beckers et al.’s study. A summary of key information regarding the studies under consideration is provided in Table 4.

Our analysis for revision surgery in Figure 2 shows a steep decline within the first year after trabeculectomy before remaining relatively stable at 80%. In contrast, the analysis for repeat surgery in Figure 3 shows only a slight decrease to 95% after the first year, with a steady decline thereafter. This suggests a preference for revision over repeat surgery within the first year after trabeculectomy. Notably, King et al.’s study aligns with our findings, highlighting that revision/bleb intervention proves to be a safe procedure and is mostly performed within the first 5 months after surgery [13]. The proportion of patients requiring revision also appears to be significantly lower in our study. Other studies have mentioned repeat surgery as a viable option for managing failed bleb filtration [14,15,16]. Our Kaplan–Meier survival analysis for success after revision (Figure 4) shows slightly lower survival rates than the initial trabeculectomy group (Figure 3). This could potentially be explained by increased scar tissue development after revision.

In our opinion, revisional surgery should usually be tried before creating a new trabeculectomy, especially within the first months following trabeculectomy. It should be the aim to spare conjunctiva for possible further surgery in the future.

While trabeculectomy remains a standard surgical procedure for glaucoma, other methods such as tube shunt surgery have since been introduced. Originally indicated for patients with a high trabeculectomy failure probability, a recent review by Bar-David et al. presented stent-based surgery as a viable first option [17].

Wagner et al. compared the surgical success of trabeculectomy with XEN implants in a retrospective study, revealing comparable success rates at 1-year follow-up (65.5% and 58.5%, respectively) [18]. However, trabeculectomy exhibited higher IOP reduction (10.5 mmHg) compared to XEN implants (7.2 mmHg).

A recent retrospective study by Lancker et al. evaluated the clinical outcome of the PreserFlo MicroShunt and compared it to trabeculectomy [19]. The complete success rates at 1 and 2 years were 55% and 40% for trabeculectomy and 60% and 55% for PreserFlo MicroShunt, respectively, stating no significant difference in survival rates between the two. However, the trabeculectomy group had a higher preoperative IOP at 25.4 mmHg (±8.5), versus 24.3 mmHg (±7.4), and a relatively short follow-up time of 19.9 months.

In another study, the Baerveldt implant was compared to trabeculectomy in terms of its efficacy [20]. The absolute success rates at 1, 2, and 3 years were 85%, 69%, and 65% for the trabeculectomy group and 83%, 60%, and 40% for the Baerveldt group, indicating better long-term performance of trabeculectomy. In addition, postoperative IOP was lower for the trabeculectomy group.

Compared to the findings from the tube shunt studies mentioned above, our study has yielded lower success rates upon follow-up after 1, 2, and 3 years (55%, 40%, and 35%). However, opposed to our study, the success criterion was set at an IOP of 21 mmHg without including medication. Also, the short follow-up time makes it difficult to assess the prolonged success of these methods.

As opposed to the devices mentioned above, which create a shunt between the anterior chamber and the subconjunctival space, the procedures discussed below bypass the trabecular meshwork.

In a study conducted by Oberfeld et al., the relative efficacy of the Hydrus Microstent in conjunction with phacoemulsification was compared with the Kahook Dual Blade (KDB) procedure [21]. At 1-year follow-up, the Hydrus Microstent demonstrated a success rate of 41.1%, accompanied by a mean IOP reduction to 14.9 mmHg (±2.8) from a baseline of 17.7 mmHg (±4.9). In comparison, the KDB procedure achieved a success rate of 42.2% and a reduction to 13.5 mmHg (±4.1) from 15.9 mmHg (±4.3).

Another surgical method bypassing the trabecular meshwork is the iStent inject device. A prospective case series on 78 eyes evaluated the surgical outcome of iStent in combination with phacoemulsification [22]. Complete success was attained at approximately 40% after 1 year and 35.1% after 2 years, with a mean follow-up time of 21.9 months and a mean preoperative IOP of 18.4 mmHg (±3.5). Complete surgical success was defined as an IOP ≤ 15 mmHg without medication.

Dang et al. investigated the long-term surgical success of the trabectome in a recent study with 120 Chinese glaucoma patients [23]. They defined surgical success as an IOP < 21 mmHg, no additional glaucoma surgery, and a >20% reduction in IOP from the baseline. They reported a success rate of approximately 80% at 1 and 2 years, with a mean preoperative IOP of 22.8 (±1.3).

Compared to the Hydrus Microstent/KDB and iStent studies, we demonstrated higher success rates. Only the iStent study had comparable success criteria, with both studies characterized by considerably lower mean preoperative IOP values and shorter follow-up periods. The trabectome studies yielded superior results. However, the milder success criteria and the lower preoperative IOP compared to our approach might contribute to these observed differences.

### Limitation of the Study

In the present study, a large number of patients with a long-term follow-up of up to 10 years were analyzed. However, a limitation of our study is its retrospective approach, which implies potential bias regarding selection and follow-up. Furthermore, our study did not include data from other clinics performing trabeculectomy, or less demanding follow-up examinations performed in an outpatient setting in private practice, which might lead to understating the long-term success of the procedure.

## 5. Conclusions

In summary, our study revealed a postoperative success rate that, while lower than reported in other studies, was achieved under notably more stringent success criteria. Furthermore, a separate analysis with milder success criteria outperformed most of the studies under consideration. Given that the present-day postoperative IOP targets are well below 21 mmHg in a clinical setting, the surgical success of trabeculectomy in the current research might be subject to overestimation. Despite the inherent invasiveness of the procedure, our findings demonstrated a low incidence of complications, underscoring the safety of trabeculectomy. Therefore, the use of stricter criteria, such as those presented in our study, should be established in order to paint a more accurate picture of the long-term efficacy of trabeculectomy. Nevertheless, our study highlights that trabeculectomy represents an effective IOP-lowering procedure.

## Figures and Tables

**Table 1:** Overview of the four different success criteria. Success was achieved when these individual criteria were fulfilled.

Overview of Success Criteria	
Criterion 1 ^1^	IOP of ≤15 mmHg and no pressure-lowering medication
Criterion 2 ^1^	No revision surgery undertaken
Criterion 3 ^1^	No IOP-lowering surgery excluding revision surgery undertaken
Criterion 4 ^2^	No IOP-lowering surgery after revision surgery undertaken

^1^ After primary TE with MMC; ^2^ after revision surgery.

**Table 2:** Descriptive statistics.

Variable	
Eyes included (*n*)	286
Age, mean	69.8 (±8.6)
Sex (female)	57%
Type of glaucoma	
POAG ^1^	217 (75.9%)
PEX ^2^	58 (20.3%)
NTG ^3^	11 (3.8%)
Preoperative IOP ^4^ in mmHg, mean	26.1 (±7.6)
Followup time in days, mean	1841 (±1567.2)
Followup time after revisional surgery, mean	1432 (±917.1)
Diabetes	26 (9%)
Anticoagulation	54 (19%)

^1^ Primary open-angle glaucoma; ^2^ pseudoexfoliative glaucoma; ^3^ normal-tension glaucoma; ^4^ intraocular pressure.

**Table 3:** Postoperative complications after trabeculectomy in total number and percentage.

Complication	*n*
Bleb leakage	30 (10.5%)
Macular edema	2 (0.7%)
Aqueous misdirection	2 (0.7%)
Hyphema	13 (4.5%)

**Table 4:** Overview of the literature and respective results.

Authors	Eyes	MFU (Years)	Pre-OP IOP (mmHg)	Success Criteria	Success Rates		
					1 Year	5 Years	MFU
Beckers et al. [7]	60	5.5	22.3	≤15/no meds	83.4%	60%	60%
Lusthaus et al. [5]	60	3	25.3	≤18 + no meds	50%	N/A	33%
Ehrnrooth et al. [8]	138	3.5	24.6	≤21 + no therapy	63%	N/A	45%
Diestelhorst et al. [11]	700	1.4	28.5	<21 + *	61%	37.8%	60%
Present study	286	5	26.1	≤15 + no meds	55%	25%	25%

* No decreased visual acuity, no increased visual field loss, and no additional glaucoma surgery. MFU—mean follow-up; Pre-OP IOP—preoperative intraocular pressure; N/A—not applicable.

## Data Availability

The data are available upon request from the corresponding author.
